# Pegbovigrastim Treatment around Parturition Enhances Postpartum Immune Response Gene Network Expression of whole Blood Leukocytes in Holstein and Simmental Cows

**DOI:** 10.3390/ani10040621

**Published:** 2020-04-03

**Authors:** Vincenzo Lopreiato, Ernesto Palma, Andrea Minuti, Juan J. Loor, Mariangela Lopreiato, Francesca Trimboli, Valeria Maria Morittu, Anna Antonella Spina, Domenico Britti, Erminio Trevisi

**Affiliations:** 1 Department of Animal Sciences, Food and Nutrition, Faculty of Agriculture, Food and Environmental Science, Università Cattolica del Sacro Cuore, 29122 Piacenza, Italy; andrea.minuti@unicatt.it (A.M.); erminio.trevisi@unicatt.it (E.T.); 2 Interdepartmental Services Centre of Veterinary for Human and Animal Health, Department of Health Science, Magna Græcia University, 88100 Catanzaro, Italy; palma@unicz.it (E.P.); trimboli@unicz.it (F.T.); spina@unicz.it (A.A.S.);; 3 Department of Animal Sciences and Division of Nutritional Sciences, University of Illinois, Urbana, IL 61801, USA; jloor@illinois.edu; 4 Department of Biochemical Sciences, Sapienza University of Rome, P.le A. Moro, 5, 00185 Rome, Italy; mariangela.lopreiato@gmail.com

**Keywords:** pegbovigrastim, Simmental, transition period, PAXgene, gene expression, immune response, leukocytes

## Abstract

**Simple Summary:**

The innate and adaptive immune system of dairy cows is impaired during the transition period, leading to an increase in susceptibility to infectious disease. Pegbovigrastim is a recombinant form of a granulocyte colony-stimulating factor that stimulates differentiation of hemopoietic stem cells to granulocytes and shortens maturation time within the bone marrow and release in circulation. The objective of the present study was to explore the effect of pegbovigrastim on whole blood leukocytes by analyzing the expression of 34 genes involved in immune and inflammatory responses immediately after calving in Simmental, a dual-purpose cow breed selected for both meat and milk production, and Holstein, a cow breed highly specialized for milk production. This study provides insight into immune cell functions impacted by pegbovigrastim treatment. Treatment of cows with pegbovigrastim increased the mRNA abundance level of most genes investigated, suggesting a thorough activation of the immune machinery during the critical post-partum period.

**Abstract:**

Pegbovigrastim is a commercial long-acting analog of bovine granulocyte colony-stimulating factor (rbG-CSF) that promotes the increased count and functionality of polymorphonuclear cells in dairy cows around the time of parturition. We hypothesized that pegbovigrastim administered to periparturient cows at approximately seven days before parturition and within 24 hours after calving could affect the profiles of gene networks involved in leukocyte function. Blood was collected on Day 3 after calving from treated groups (pegbovigrastim (PEG); 13 Simmental (seven multiparous and six primiparous) and 13 Holstein (seven multiparous and six primiparous) cows) that received pegbovigrastim (Imrestor; Elanco Animal Health) and controls (CTR; 13 Simmental (seven multiparous and six primiparous) and 13 Holstein (six multiparous and seven primiparous) cows) that received saline solution. Blood from all cows was sampled from the jugular vein in a PAXgene Blood RNA System tube (Preanalytix, Hombrechtikon, Switzerland) for RNA extraction. The RT-qPCR analysis was performed to investigate a panel of 34 genes of interest, representing recognition, immune mediation, migration, cell adhesion, antimicrobial strategies, inflammatory cascade, oxidative pattern, and leukotrienes in whole blood leukocytes. Normalized data were subjected to the MIXED model of SAS (ver. 9.4) with treatment, breed, parity, and their interaction as fixed effects. Compared with CTR, whole blood leukocytes of PEG cows had higher expression of genes involved in recognition and immune modulation (*CD14*, *CD16*, *MYD88*, *TLR2*, and *TLR4*), cell adhesion (*ITGB2*, *ITGAL*, *TLN1*, *SELL*, *SELPLG*, and *CD44*), antimicrobial activity (*MMP9*, *LTF*, and *LCN2*), and inflammatory cascade (*CASP1*, *TNFRSF1A*, *IL1B*, *IL1R*, *IL18*, *IRAK1*, *NLRP3*, and *S100A8*). This suggested an improvement of migration, adhesion, and antimicrobial ability and an enhanced inflammatory response, which in turn could trigger immune cell activation and enhance function. Expression of *SOD2* and *ALOX5* was also greater in the PEG group. In contrast, compared with CTR cows, PEG led to lower expression of *RPL13A*, *ALOX15*, *IL8*, and *TNF*. Overall, leukocytes from Simmental compared with Holstein cows had greater expression of *IDO1*, *RPL13A*, *ALOX5*, *CD44*, *CX3CR1*, *ITGB2*, and *TNFA*, whereas expression of *CD16* and *TLR2* was lower. Overall, compared with multiparous cows, primiparous cows had higher expression of *IL1B*, *IL18*, *MYD88*, *SELL*, and *TLR2* and lower expression of *MMP9*. Simmental cows seemed more sensitive to induction of the immune system after calving, as revealed by the greater abundance of genes involved in immune system adaptation, regardless of pegbovigrastim treatment. Primiparous cows undergoing a new stress condition with respect to older cows were characterized by leukocytes with a higher inflammatory response. In conclusion, pegbovigrastim led to higher expression levels of most genes involved in the processes investigated, suggesting a thorough activation of the immune machinery during the critical post-partum period.

## 1. Introduction

The negative energy balance of dairy cows during the transition period is associated with reduced immune function and increased concentrations of some blood metabolites due to tissue mobilization [1,2]. Polymorphonuclear neutrophil (PMN) and lymphocyte functions decrease gradually starting about 2 wk before calving, with the lowest efficiencies between time of calving and two days after [3,4]. Together, these metabolic and immunologic challenges that occur during the peripartal period are important factors that limit the ability of most cows to achieve optimal performance and immune-metabolic status [5,6]. In order to improve immune efficiencies around parturition, a long-acting recombinant bovine granulocyte colony-stimulating factor (rbG-CSF; pegbovigrastim, Imrestor, Elanco Animal Health, Greenfield, IN) was recently developed. The colony stimulating factor is normally produced by a variety of cells including monocytes, macrophages, and cells of mesodermal origin including vascular endothelial cells, fibroblasts, and keratinocytes [7,8]. It induces differentiation of progenitor cells into mature neutrophils, shortens maturation time within the bone marrow, and alters functionality by increasing phagocytosis and antibody-dependent cell-mediated cytotoxicity [9]. Previous studies reported that pegbovigrastim treatment increased PMN number and improved bacteria engulfment and cytotoxicity by PMN in periparturient Holstein cows [10,11]. 

Application of transcriptomics tools have enhanced our understanding of the molecular mechanisms controlling the basic functions of immune cells during stressful periods [12,13]. Crookenden et al. [14] reported an altered inflammatory gene expression response in neutrophils during the transition period, which confirmed evidence of changes in PMN function in response to calving. In this respect, Heiser et al. [15] hypothesized that pegbovigrastim could have affected gene expression networks of neutrophils supporting their functions during the periparturient period. Indeed, the authors reported a greater abundance of genes involved in migration, pattern recognition, and inflammatory response. In addition, pegbovigrastim also affected RNA expression in uterine cells of genes involved in cell adhesion, recognition, inflammation, and antimicrobial functions, suggesting greater ability of neutrophils to migrate into their target tissues. It is noteworthy that studies on immune dysfunction and impairment of neutrophil function during the transition period have been undertaken using high-yielding dairy cows or cows in pasture-based systems, but only using Holsteins. Thus, the objective of the present study was to explore the effects of pegbovigrastim on whole blood leukocyte gene networks by analyzing the transcription profiles of 34 genes involved in recognition, immune mediation functions, migration, cell adhesion, antimicrobial strategies, inflammatory cascade, oxidative stress, and leukotriene production from Simmental and Holstein cows immediately after calving. 

Furthermore, we previously provided evidence of unique expression marks immediately after parturition between immune cells of Simmental cows and Holstein cows [16]. Hence, we hypothesized that leukocyte gene expression response after administration of pegbovigrastim would differ between cows highly specialized for milk production, Holsteins, and a dual-purpose cow breed selected for both meat and milk production, such as Simmentals.

## 2. Materials and Methods 

### 2.1. Animals, Treatments, and PAXgene Blood Sampling

The trial was carried out in accordance with Italian laws on animal experimentation (DL n. 26, 04/03/2014) and ethics (Authorization of Italian Health Ministry N 403/2017-PR). The experimental design involved 26 Simmental (14 multiparous and 12 primiparous) and 26 Holstein cows (13 multiparous and 13 primiparous). Details of management, diet, health status, and metabolic status have been published previously [17]. Briefly, management procedures for Simmental and Holstein cows were similar: cows were fed a total mixed ration (TMR) once daily (0700 and 0800 h, lactating and dry cows, respectively) and after parturition milked twice daily (at 0500 and 1700 h). Once enrolled in the study, cows were alternately assigned into 2 groups based on expected calving date and parity: treated group (pegbovigrastim (PEG); 13 Simmental (7 multiparous and 6 primiparous) and 13 Holstein (7 multiparous and 6 primiparous) cows) that received pegbovigrastim (Imrestor; Elanco Animal Health) and control group (CTR; 13 Simmental (7 multiparous and 6 primiparous) and 13 Holstein (6 multiparous and 7 primiparous) cows) that received saline solution. Treatments were administered via subcutaneous injection in the scapular region using prefilled syringes, provided by the manufacturer, for PEG (15 mg of pegbovigrastim in 2.7 mL solution) and syringes with an 18 gauge × 2.5-cm needle for CTR (2.7 mL of sterile saline). The first dose was administrated approximately 7 days before expected parturition, depending on the judgement of investigators based on physical changes including swelling of vulva and filling of udder. The second dose was administrated within 24 h after calving. On Day 3 after calving, blood was sampled from the jugular vein into PAXgene Blood RNA System tubes (Preanalytix, Hombrechtikon, Switzerland) for RNA extraction. The use of PAXgene tubes in cattle has been reported previously [16,18,19].

### 2.2. RNA Extraction, cDNA Synthesis, Target Genes, and Quantitative PCR

The complete procedure for RNA extraction from PAXgene RNA tubes was performed following the manufacturer’s protocol (Blood RNA Kit Handbook, PreAnalitix GmbH, Quiagen, Hilden, Germany), and the protocol used for cDNA synthesis was exactly the same as that reported by Lopreiato et al. [16]. Briefly, the reaction was performed in an Eppendorf Mastercycler^®^ Gradient (Eppendorf, Hamburg, Germany) using the following temperature program: 25 °C for 5 min, 42 °C for 60 min, and 70 °C for 5 min. The resulting cDNA was diluted 1:4 (v:v) with DNase/RNase-free water and stored at −80 °C until qPCR reaction. Genes under investigation selected for transcript analysis were those related to recognition and immune mediation functions (*CD14*, *CD16*, *MYD88*, *TLR2,* and *TLR4*), migration and cell adhesion (*CCR2*, *CX3CR1*, *ITGB2*, *ITGAL*, *TLN1*, *SELL*, *SELPLG*, *CD44*, and *LGALS8*), antimicrobial strategies (*MMP9*, *LTF*, *MPO*, *LCN2*, and *IDO1*), oxidative stress (*SOD1* and *SOD2*), leukotriene function (*ALOX5* and *ALOX15*), and genes related to the inflammatory cascade (*CASP1*, *TNFRSF1A*, *IL1B*, *IL1R*, *IL8*, *IL18*, *IRAK1*, *TNF*, *NLRP3*, *S100A8*, and *RPL13A*). The complete procedure for the PCR primers’ validation and information was published elsewhere [16]. The qPCR was performed in an Optical 384-Well Reaction Plate (CFX384 Touch; Bio-Rad, Hercules, CA, USA). Complete details of the procedure were previously reported by Lopreiato et al. [16]. 

### 2.3. RT-PCR Data Analysis

The qPCR efficiency and quantification cycle values were obtained for each reaction using LinRegPCR (Version 2017.1), a program for the analysis of quantitative RT-PCR (qPCR) data resulting from monitoring the PCR reaction with SYBR green or similar fluorescent dyes. The program determines a baseline fluorescence and carries out a baseline subtraction. Then, a window-of-linearity was set, and PCR efficiencies per sample were calculated. With the mean PCR efficiency per amplicon, the Cq value per sample, the fluorescence threshold (set to determine the Cq), and the starting concentration per sample (expressed in arbitrary fluorescence units) were calculated. The final data were normalized using the geometric mean of 3 internal control genes: *ACTB*, *SDHA*, and *YWHAZ*. These internal control genes were previously confirmed as suitable for PMNL gene expression analysis [13,14,16]. Furthermore, the stability of the normalization factor of these 3 endogenous control genes was assessed using GeNorm software with a favorable final pairwise variation of 0.11, and no improvement was obtained in stability with the addition of a fourth endogenous control gene.

### 2.4. Statistical Analysis

Statistical analysis was performed using ANOVA in the MIXED procedure of SAS Version 9.4 (SAS Institute Inc., Cary, NC) according to the following model:

*Y*_*ijkl*_ = *μ* + *B*_*i*_ + *G*_*j*_ + *P*_*k*_ + *BG*_ij_ + *GP*_*jk*_ + *BGP*_*ijk*_ + *c*_*m:ij*_ + *ε*_*ijkl;*_ (1)

where *Y_ijkl_* = dependent continuous variable, *μ* = overall mean, *B_i_* = fixed effect of breed (*i* = Simmental vs. Holstein), *G_j_* = fixed effect of treatment (*j* = PEG vs. CTR), *P_k_* = fixed effect of parity (primiparous vs. multiparous), *BG_ij_* = interaction between breed and treatment, *GP_jk_* = interaction between treatment and parity, *BGT_ijk_* = interaction among breed, treatment, and parity, *c_m:ij_* = random effect of the *m*^th^ animal (cow) nested within breed × treatment × parity, and *ε_ijkl_* = residual error. The Kenward–Roger statement was used for computing the denominator degrees of freedom. The normality of the data was checked with the Univariate procedure of SAS (ver. 9.4). Variables not normally distributed were log10 transformed. Asterisks in tables with results indicate variables that were transformed. Data were considered significant at *p* ≤ 0.05 using the *p*-values for differences (PDIFF) statement in SAS (ver. 9.4).

## 3. Results

The first dose was administered at approximately 7 d (on average, 7.80 ± 5.50 d) before expected parturition, depending on the judgment of the investigators, based on physical changes including swelling of vulva and filling of udder. The second dose was administered within 24 h after calving.

Genes were grouped into functional pathways (Table 1, Table 2, Table 3, Table 4 and Table 5) and reported as least squared means (LSM) of arbitrary mRNA abundance with the standard error of the mean (SEM) and the significance of group, breed, parity, and their interaction effect (*p*-value). The interaction of breed × parity was not taken into account in the current study because we focused on the main effects alone and their interaction with treatment only.

### 3.1. Expression of Genes Involved in the TLR Pathway and Immune Mediation

Expression of genes involved in toll-like receptor (TLR) signaling, *TLR2*, *TLR4*, *MYD88*, and *CD14*, was greater in PEG cows compared with CTR cows (*p* < 0.05; Table 1). Similarly, CD16 expression (involved in the removal of the antigen-antibody complex from the circulation) was greater in PEG cows compared with CTR cows (*p* < 0.05; Table 1). Overall, leukocytes of Holstein cows were characterized by a greater abundance of *TLR2*, which also was higher in primiparous cows (*p* < 0.05; Table 1). Furthermore, a main effect of parity was detected for *MYD88* expression resulting from higher values in primiparous compared with multiparous cows (*p* < 0.05; Table 1). 

### 3.2. Expression of Genes Involved in Cell Migration and Adhesion

**Figure 1:**
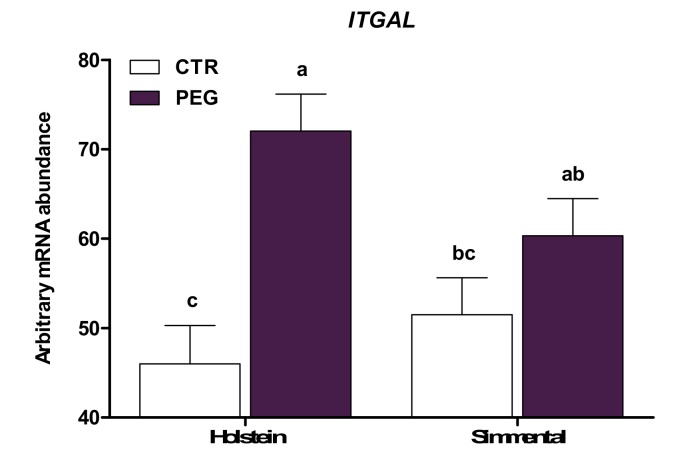
LSM ± SEM of arbitrary mRNA abundance for gene expression of *ITGAL* (integrin subunit alpha L) evaluated three days after calving in Simmental and Holstein cows treated either with pegbovigrastim (PEG) or with saline (CTR) at approximately Day 7 relative to calving and on the day of calving within 24 h. Different letters represent significant differences among groups at *p* ≤ 0.05.

**Figure 2:**
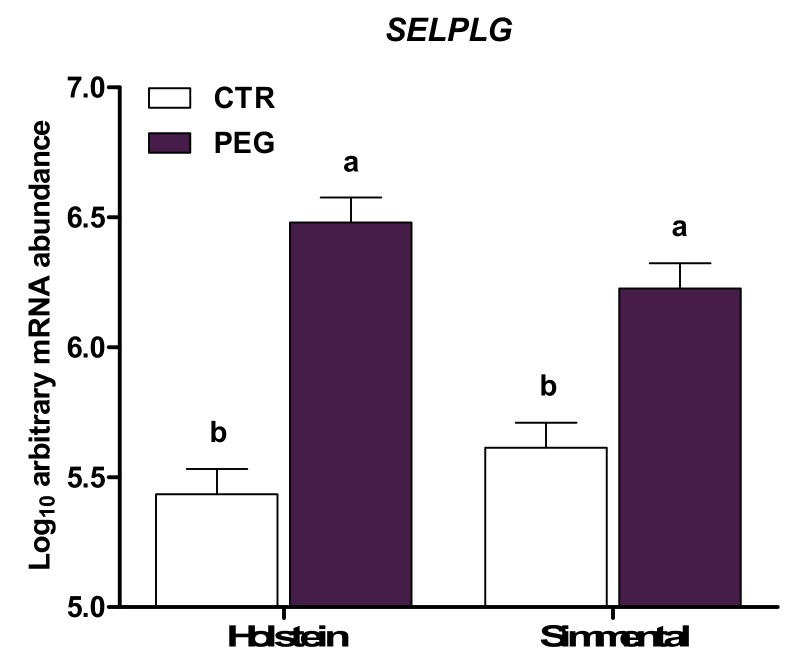
LSM ± SEM of Log10 arbitrary mRNA abundance for gene expression of *SELPLG* (selectin P ligand) evaluated three days after calving in Simmental and Holstein cows treated either with pegbovigrastim (PEG) or with saline (CTR) at approximately Day 7 relative to calving and on the day of calving within 24 h. Different letters represent significant differences among groups at *p* ≤ 0.05.

The expression of genes involved in migration and adhesion of immune cells is presented in Table 2. *CCR2* and *CX3CR1*, involved in cell chemotaxis, were not affected by pegbovigrastim (*p* > 0.10). Treatment with pegbovigrastim led to greater expression of genes encoding integrins (*ITGB2* and *ITGAL*), adhesion molecules (*TLN1*), selectin (*SELL*), and selectin ligands (*SELPLG* and *CD44*) compared with CTR cows (*p* < 0.01). A main effect of breed was detected for *CX3CR1*, *ITGB2*, and *CD44*, due to upregulation in Simmental (*p* ≤ 0.05) compared with Holstein cows. In addition, a group × breed interaction was observed for *ITGAL* expression. Leukocytes from Holstein PEG cows had higher expression of *ITGAL* compared with Holstein CTR (*p* < 0.05; Figure 1), whereas no differences were observed in Simmental cows (*p* > 0.05). The expression of *SELPLG* was also affected by group × breed. However, the post-hoc test pointed out no differences between Simmental and Holstein CTR cows and between Simmental and Holstein PEG cows, but a higher expression in PEG cows of both Simmental and Holstein cows (*p* < 0.05; Figure 2) was obtained, compared with CTR cows of both Simmental and Holstein. Overall, compared with multiparous, leukocytes of primiparous cows had greater expression of *SELL* (*p* = 0.05).

### 3.3. Expression of Genes Involved in Antimicrobial Functions

**Figure 3:**
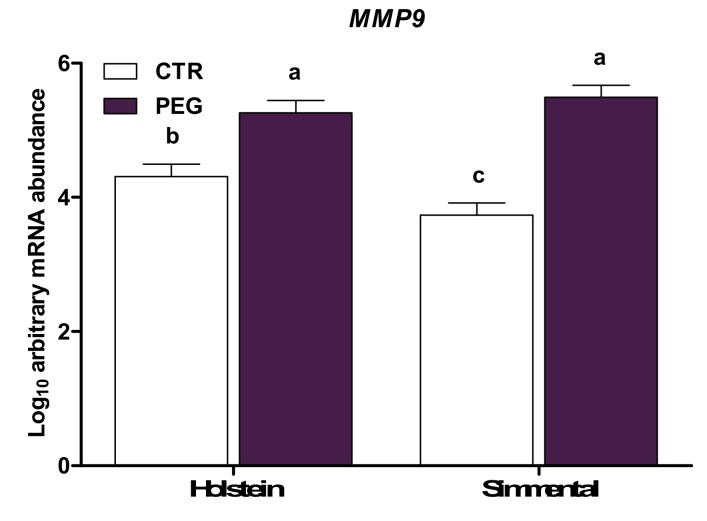
LSM ± SEM of Log10 arbitrary mRNA abundance for gene expression of *MMP9* (matrix metallopeptidase 9) evaluated three days after calving in Simmental and Holstein cows treated either with pegbovigrastim (PEG) or with saline (CTR) at approximately Day 7 relative to calving and on the day of calving within 24 h. Different letters represent significant differences among groups at *p* ≤ 0.05.

**Figure 4:**
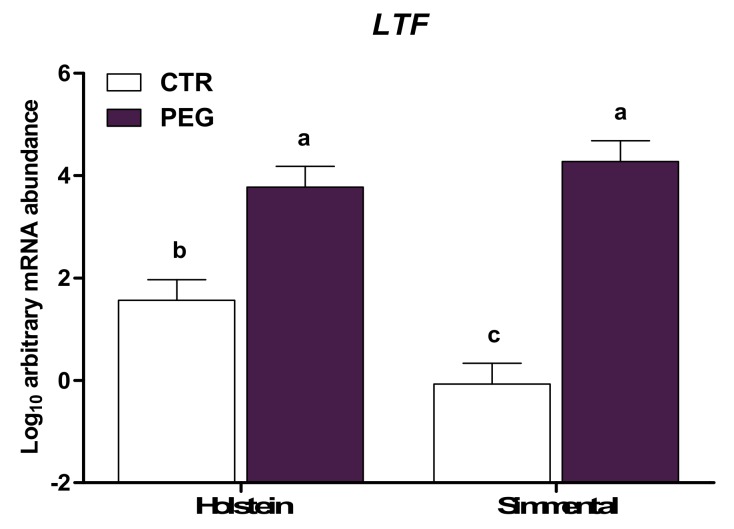
LSM ± SEM of Log10 arbitrary mRNA abundance for gene expression of *LTF* (lactotransferrin) evaluated three days after calving in Simmental and Holstein cows treated either with pegbovigrastim (PEG) or with saline (CTR) at approximately Day 7 relative to calving and on the day of calving within 24 h. Different letters represent significant differences among groups at *p* ≤ 0.05.

**Figure 5:**
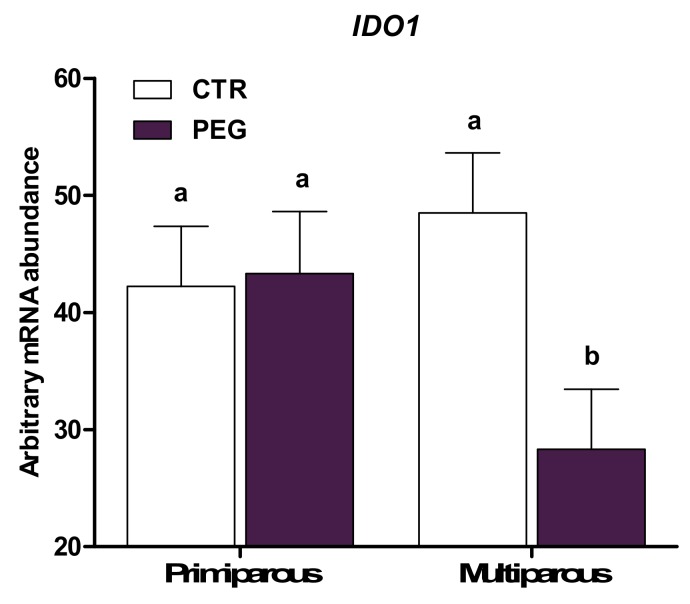
LSM ± SEM of Log10 arbitrary mRNA abundance for gene expression of *IDO1* (indoleamine 2,3-dioxygenase 1) evaluated three days after calving in primiparous and multiparous cows treated either with pegbovigrastim (PEG) or with saline (CTR) at approximately Day 7 relative to calving and on the day of calving within 24 h. Different letters represent significant differences among groups at *p* ≤ 0.05.

Compared with CTR, whole blood leukocytes from cows subjected to pegbovigrastim had higher expression of *MMP9*, *LTF*, *MPO*, and *LCN2* of the five genes evaluated that are involved in the antimicrobial functions (*p* < 0.01; Table 3). In particular, expression of *MMP9* and *LTF* was lower in Simmental CTR compared with Holstein CTR (group × breed, *p* < 0.05; Figure 3 and Figure 4, respectively). However, Simmental PEG displayed a similar expression of *MMP9* and *LTF* compared with Holstein PEG cows (*p* > 0.05), but higher expression compared with CTR cows for both Simmental and Holstein (*p* < 0.01). Overall, multiparous cows had higher expression of *MMP9* compared with primiparous (*p* < 0.05). An interaction group × parity effect was detected for *IDO1* (*p* < 0.05; Figure 5), where pegbovigrastim caused a lower abundance in multiparous PEG cows compared with multiparous CTR cows. In addition, the expression of *IDO1* was lower in multiparous PEG cows compared with primiparous PEG cows (*p* > 0.05).

### 3.4. Expression of Genes Involved in the Inflammatory Response

**Figure 6:**
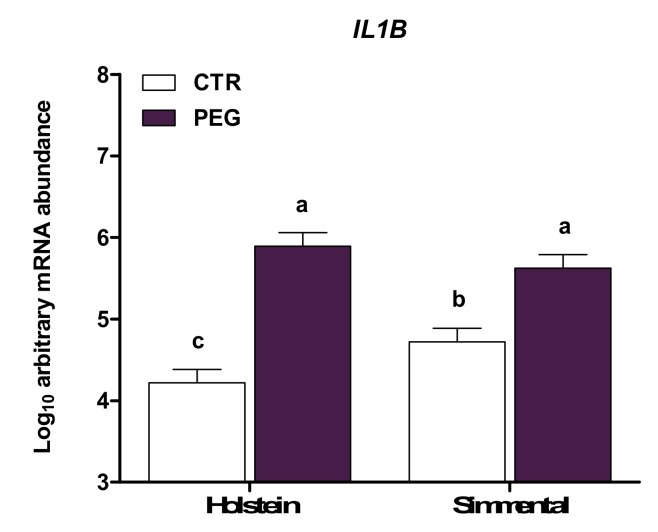
LSM ± SEM of Log10 arbitrary mRNA abundance for gene expression of *IL1B* (interleukin 1 beta) evaluated three days after calving in Simmental and Holstein cows treated either with pegbovigrastim (PEG) or with saline (CTR) at approximately Day 7 relative to calving and on the day of calving within 24 h. Different letters represent significant differences among groups at *p* ≤ 0.05.

Table 4 reports the expression of genes involved in the inflammatory pattern. As a result of pegbovigrastim treatment, compared with control cows, expression of *CASP1*, *TNFRSF1A*, *IL1B*, *IL1R*, *IL18*, *IRAK1*, *NLRP3*, and *S100A8* was higher (*p* < 0.05). In contrast, *IL8*, *TNF*, and *RPL13A* abundance was lowered by pegbovigrastim (*p* < 0.05). Specifically, *IL1B* and *IL18* expression was affected by parity and only for *IL1B* by group × breed effects. Primiparous cows had higher *IL1B* and *IL18* expression compared with multiparous (*p* > 0.05), whereas Holstein CTR cows had lower *IL1B* expression compared with Simmental CTR (*p* < 0.05; Figure 6). Overall, leukocytes of Simmental cows had higher expression of *TNF* and *RPL13A* compared with Holstein cows (Breed, *p* < 0.05). 

### 3.5. Expression of Genes Involved in the Oxidative Stress and Leukotrienes

Regarding genes involved in oxidative stress, only *SOD2* expression was greater in PEG compared with CTR cows (*p* < 0.01; Table 5). Expression of genes involved in leukotriene synthesis (*ALOX5* and *ALOX15*) had opposite responses in cows treated with pegbovigrastim (Table 5). For instance, *ALOX5* abundance was greater, whereas *ALOX15* abundance was lower (*p* < 0.05) in PEG compared with CTR cows. Overall, Simmental cows displayed a greater expression of *ALOX5* compared with Holstein cows (*p* < 0.05).

## 4. Discussion

Previous studies from our group provided evidence of a different immunometabolic response between immune cells of Simmental and Holstein cows immediately after parturition [16,17]. Thus, modulation of those genes involved in immune adaptation and inflammatory response are breed-specific and also responsive to pegbovigrastim. 

In human, G-CSF is known to influence immune responses modulating immune cell composition, cytokine profiles, immune cell responses, and inflammatory response [20]. Pegbovigrastim is a recombinant bovine G-CSF and was shown to increase the concentrations of circulating mature PMN in the blood and their relative immune function in periparturient dairy cows [21]. To date, only one study evaluated the effect of pegbovigrastim on inflammatory, cell adhesion, and pattern recognition genes in pasture-fed periparturient Holstein and Jersey cows [15].

As evidenced by the higher abundance of all genes investigated (*CD14*, *CD16*, *MYD88*, *TLR2*, and *TLR4*), immune-related gene expression changes occurring after pegbovigrastim administration revealed activation of recognition and immune-mediation signaling. Toll-like receptors (TLRs) are pivotal during the innate immune response against pathogens, because they recognize foreign non-self-material and trigger the innate inflammatory through the activation of NF-κB, which in turn leads to the transcription of an array of inflammatory cytokine genes [22]. Hence, expression of an increased number of pattern recognition receptors and their transduction adapters (such as MYD88) is likely to be beneficial. In fact, their signaling rapidly induces various inflammatory cytokines, chemokines, and type I interferons, consequently triggering an array of antimicrobial immune responses [15,23]. The topic-review of Spiekermann et al. [24] summarized several in vitro, as well as human clinical trial outcomes to report that G-CSF and GM-CSF enhance the ability of neutrophils to eliminate microbial organisms. In this regard, TLRs and CD14 play an essential role in mediating this cellular response to a large array of microbial ligands. Our results were in agreement with those reported by Kurt-Jones et al. [25], who found upregulation, in terms of gene transcription and surface protein expression, of TLR2 and CD14 levels in neutrophils and monocytes. 

As a consequence of greater levels for relative mRNA of *CD14*, *CD16*, *MYD88*, *TLR2*, and *TLR4*, an overall pro-inflammatory response was stimulated by pegbovigrastim treatment as revealed by greater abundance of *TNFRSF1A*, *IL18*, *IL1R*, *IRAK1, CASP1,* and *IL1B,* compared with control animals. This outcome confirmed the role of G-CSF in both bovine and humans [26]. In the Supplementary File are reported the KEGG Toll-like receptors (Figure S1) and NF-kappa B (Figure S3) signaling pathways, showing how pegbovigrastim affected the genes investigated and how they signal down-stream. The interleukin-1 (IL-1) family of cytokines and receptors is important in immunology because the IL-1 family and Toll-like receptor (TLR) families share similar functions. More than any other cytokine family, the IL-1 family is primarily associated with innate immunity [27]. Furthermore, proinflammatory cytokines such as IL-1β and TNF-α are key mediators of cell communication within the inflammatory angiogenic area, and they positively regulate the synthesis of proangiogenic factors [27]. In our study, from the simultaneous increase of cytokine and cytokine receptor-related genes (*IL1B*, *IL1R*, and *TNFRSF1A*), which act as checkpoint regulators for the recruitment, trafficking, and maturation of leukocytes during the inflammatory response [15,28], we can speculate that this increment was a reflection of a higher number and a better primary response of the immune cells. All these signaling pathways result in cellular production of proinflammatory cytokines, but if the efficiency of this signal is compromised, there could be repercussions on the production and on expression of proinflammatory cytokines. In fact, *TNF* and *IL8* expression was lower after treatment, counteracting in part the inflammation process. In this regard, it is noteworthy to report that the lower expression of *TNF* and *IL8* was consistent with the study of Fukuzono et al. [29]. From human peripheral blood neutrophils, the authors found that G-CSF inhibited LPS-induced IL-8 and TNF-α production, likely via the activation of JAK2/STAT3 (Janus kinase 2/signal transducer and activator of transcription 3), which may be involved in the suppressive effect of G-CSF on these cytokines’ production in human neutrophils. To support the latter, using immortalized mouse macrophages and human monocytic cell line (THP-1), Kim et al. [30] demonstrated that the suppression of LPS-induced TNF-α production by G-CSF was mediated through activation of STAT3 and subsequent inhibition of c-Jun-N-terminal kinases’ (JNKs) activation. The exact mechanism by which G-CSF enhances the gene regulation of some pro-inflammatory cytokines and diminishes others remains poorly understood. 

Besides the mRNA increase of TLRs’ gene network, pegbovigrastim treatment increased *NLRP3* and *CASP1* mRNA relative abundance. These genes encode respectively for the NOD-like receptor protein 3 inflammasome and caspase-1 (or interleukine-1 converting enzyme). The intracellular NOD-like receptor (NLR) family plays a pivotal role in the recognition of intracellular ligands. NOD1 and NOD2, two prototypic NLRs, sense the cytosolic presence of the bacterial peptidoglycan fragments that escape from endosomal compartments, driving the activation of NF-kappa B and MAPK, cytokine production, and apoptosis. On the other hand, a different set of NLRs induces caspase-1 activation through the assembly of multiprotein complexes called inflammasomes. The activated of caspase-1 regulates maturation of the pro-inflammatory cytokines IL-1β and IL-18 [31]. These reviewed findings supported our results, where, from Figure S2 of the Supplementary File, it is clearly evident that pegbovigrastim also “activated” the inflammasome, which in turn led to a down-stream increase, coupled with signals from TLRs pathway, of *IL1B* and *IL18* mRNA abundance. Overall, our findings highlight the role of pegbovigrastim in orchestrating inflammatory response with direct down-stream regulation of related genes (NOD-like receptor signaling pathway, Toll-like receptor signaling pathway, and NF-kappa B signaling pathway).

Overall, pegbovigrastim enhanced leukocyte migration and adhesion gene network expression, as demonstrated by the greater abundance of most genes related to these processes (Figure S4 of the Supplementary File). LFA-1, lymphocyte function-associated antigen-1, is one of the beta-2-integrins, a family of adhesion molecules that mediate firm attachment of mainly lymphocytes, but also monocytes and neutrophils [32] to endothelial cells following the initial rolling and tethering step [33]. This integrin is formed by the integrin alpha L chain (ITGAL) combined with the beta 2 chain (ITGB2), whose gene expressions were increased after pegbovigrastim treatment. Since LFA-1 is an important accessory adhesion molecule necessary for efficient extravasation of lymphocytes, monocytes, and neutrophils from blood into tissue [34], the increased gene expression of *ITGAL* and *ITGB2* genes in PEG cows compared with CTR may be related to a higher response of these cells to increase the recruitment into tissue, mainly mammary gland and uterus, which are the most challenged after parturition. The activation of *SELPLG* and *SELL* genes plays a critical role in leukocyte trafficking during inflammation. In our study, *SELPLG* and *SELL* mRNA expressions were higher in both breeds of the PEG group. Contrary to the current findings, Heiser et al. [15] did not observe any influence by pegbovigrastim on migration and cell adhesion gene expression. We hypothesized that the type of immune cell population examined may have affected the outcomes in both studies. Indeed, Heiser et al. [15] isolated neutrophils from blood, whereas the present study took into account the mRNA expression from the total leukocyte population. Furthermore, in both Holstein and Simmental cows, we observed a higher abundance *MMP9*, in leukocytes of PEG cows. MMP9 is a collagenase that belongs to the gelatinase B group [35], a family of MMPs capable of degrading at least one component of the extracellular matrix or basement membrane. This capability aids in the migration of white blood cells from blood to the site of inflammation. MMP9 is stored in tertiary granules within the bovine neutrophils and is released when neutrophil degranulation is induced by either chemical or microbial stimuli. After release, MMP stimulates neutrophil migration [35]. Hence, leukocyte migration across extracellular matrix proteins is dependent on matrix degradation, not only for facilitating “matrix permeability”, but also for generating extracellular matrix-derived fragments, which are biologically active and can be highly chemotactic for leukocytes [36]. The increase of *MMP9* mRNA abundance in PEG cows, thus, is consistent with the greater mRNA levels of *ITGAL*, *ITGB2*, *SELPLG*, and *SELL* involved in the transendothelial migration of leukocytes (Figure S4). In this respect, pegbograstim treatment was also able to regulate and coordinate the transcription of these pivotal genes, allowing for the migration of leukocytes once relative proteins are properly expressed.

The main effect of breed highlighted the greater abundance of *CX3CR1* and *CD44* in Simmental compared with Holstein cows. This may be indicative of greater migration and chemotaxis. Chemokine receptors are pivotal for chemotaxis and are essential for immune cell migration during bacterial infection [16]. The CX3CR1 protein is the receptor for CX3CL1, also known as fractalkine. CX3CR1 and its interaction with CX3CL1 mediate chemotaxis of immune cells during inflammatory response [37]. In addition, CD44 was identified as one of three endothelial-selectin (E-selectin) ligands on PMNL; in fact, CD44 plays an important role in the mediation and control of blood PMN recruitment in response to inflammatory signals [38]. Together, the greater abundance of *ITGAL*, *CD44*, and *CX3CR1* after calving suggested a potential greater influx and adhesion of leukocytes into tissues, likely contributing to decrease the incidence of infection postpartum. Pegbovigrastim treatment enhanced the entire repertoire of genes involved in chemotaxis, cell-cell interaction, and migration, providing evidence of its effect in boosting the regulation of adhesion and migration capacity even after parturition, when cows are challenged by an impairment of gene expression related to the inflammatory and immune response in circulating neutrophils [14].

Pegbovigrastim increased also the mRNA expression of those genes involved in the antimicrobial activity, *LTF*, *MPO, LCN2,* and *IDO1* in both breeds. When released, MPO causes neutrophils to kill invading pathogens directly by phagocytosis or by the release of antimicrobials [39]. MPO synthesis is mainly limited to myeloid cells in the bone marrow. The influence of pegbovigrastim on MPO expression, an enzyme stored in azurophilic granules of granulocytes, agreed with increased bone marrow production induced by treatment in the study of Trimboli et al. [40]. In this case, our results were opposite those reported by Heiser et al. [15] for the same gene, i.e., pegbovigrastim did not upregulate *MPO*. However, the study of Heiser et al. [15] led to a small, but significant increase of *MPO* expression in uterine cells. IDO1 (indoleamine 2,3-dioxygenase), other than mediating immunosuppression, displays antimicrobial and antiviral effects by reducing the availability of the essential amino acid tryptophan in the inflammatory environment [41]. In addition, in pregnancy, *IDO1* expression in endothelial cells may inhibit extravasation of microbial pathogens from the blood into the tissue through the depletion of tryptophan in the local tissue adjacent to the blood stream, and this may contribute to protection of the fetus against infection [41]. In our study, there was no effect of PEG on *IDO1*. However, in PEG Simmental cows, the levels of *IDO1* were lower than CTR Simmentals, and in multiparous cows, pegbovigrastim led to lower abundance compared with control cows. The lack of effect of PEG on *IDO1* was also confirmed in human studies [42,43], where G-CSF was not able to induce the activation of IDO1 directly. However, this outcome should be further clarified in future studies. In particular, neutrophil maturation is characterized by progressive acquisition of the morphologic, biochemical, and functional features that distinguish the mature neutrophil from other leukocytes. The transition from primitive myeloblast to promyelocyte is marked by the acquisition of primary (“azurophilic” or “nonspecific”) granules, whereas the acquisition of secondary (“neutrophilic” or “specific”) granules accompanies the transition from promyelocyte to myelocyte. Myelocytes exhibit features of the terminal stages of maturation: proliferative capacity is reduced; specific “mature” functions such as phagocytosis and bacterial killing become apparent; and maturation along other granulocyte-monocyte pathways is no longer possible. The acquisition of secondary granules can thus be regarded as a major event in this terminal process [44,45]. In this respect, Rado et al. [44] suggested that lactoferrin (involved in several physiological and protective functions, including regulation of iron absorption in the bowel and mainly antimicrobial activities [46]) is synthetized in developing neutrophils during the maturation process and present in secondary granules of PMNs. Thus, the latter could imply that pegbovigrastim was also able to induce neutrophils’ maturation (supported also by the greater levels of *MPO* and *LCN2*), besides the effect of stimulating the formation of granulocyte colonies from bone marrow-derived precursors [40].

Oxidative stress occurs when an imbalance exists between the production of reactive oxygen metabolites and the neutralizing availability of antioxidants [47]. These conditions can contribute and/or lead to the onset of health disorders in cattle, in particular during the transition period [48]. In fact, it has been observed that during the transition period, cows can experience oxidative stress [49], which may exacerbate metabolic disease incidence [47]. In this study, we examined for the first time the effect of pegbovigrastim on oxidative-related genes. *SOD2* abundance was greater in the PEG group without differences between the two breeds or between primiparous and multiparous cows. In the study of Crookenden et al. [14], *SOD1* expression was lowest at −1 wk, increased on the day of calving, and remained elevated across the 4-wk measurement period. In other recent observations on the oxidative burst following G-CSF treatment in periparturient dairy cows, no significant differences were noted between treatment groups in the release of superoxide anion (oxidative burst) or phagocytosis by PMN during the experimental period [10]. In contrast, others reported a decrease in oxidative burst in the periparturient period [50]. 

Besides cytokines and chemokines, lipid mediators such as leukotrienes trigger an inflammatory response to attract leukocytes to sites of bacterial infection [51]. The release of arachidonic acid from membrane phospholipids is the first step in the production of leukotrienes, and ALOX5 mediates the conversion of arachidonic acid to 5(S)-hydroperoxy-6-trans-8,11,14-cis-eicosatetraenoic acid, and further to the allylic epoxide 5(S)-trans-7,9-trans-11,14-cis-eicosatetrenoic acid (leukotriene A_4_). Leukotrienes promote leukocyte recruitment and enhance leukocyte respiratory burst activity, as well as cytokine production [52,53,54]. In particular, leukotriene B_4_ has been reported to be among the most potent endogenous chemoattractants, and its formation is derived via leukotriene A_4_ hydrolysis [55]. Thus, the greater abundance of *ALOX5* in PEG cows derives from the higher expression of cytokine-related genes (especially *IL1B* and *TNF*), confirming the greater sustained immune response of leukocytes from PEG compared with CTR cows. Unlike *ALOX5*, the expression of *ALOX15* in PEG cows was lower compared with CTR cows. 15-lipoxygenase-1 (ALOX15) plays an important role in the formation of key lipid mediators (e.g., lipoxins and resolvins) to terminate inflammation [56]. Hence, the greater abundance of *ALOX5* and lower abundance of *ALOX15* in PEG cows likely helped sustain the peculiar inflammatory response after pegbovigrastim treatment, which in turn supported immune cell activation and functionality, especially after calving when immune dysregulation occurs. 

Therefore, with the exception of *IL8*, *TNF*, and *ALOX15*, in blood leukocytes, the inflammatory cascade resulted in a greater abundance of all genes involved (including *ALOX5*). This agreed with the fact that pegbovigrastim was able to induce differentiation of myeloid cells into mature granulocytes, which in turn triggered the complex mechanisms of interaction of immune cells with the consequent recruitment of other leukocytes. This response is also supported by the highest expression of antimicrobial activity genes (except for *IDO1*).

## 5. Study Limitation

Gene expression profiling has emerged as an important and essential tool to detect any primary response of cells to challenges. The immune system represents the first barrier acting to counteract environmental damages to which cells could be exposed. Hence, gene expression analyses provide information about which genes are expressed, down- or up-regulated, at any specific given time. Nevertheless, by its nature, gene expression profiling does not give information regarding the functional activities of the protein encoded, considering also the post-translational modifications occurring in all living cells, which in turn regulate protein functions. The aim of the present work was to gain insights into the first cellular events that characterize the response of the immune system, impaired in dairy cows during the transition period, upon pegbovigrastim stimulation. We focused on 34 genes of the immune and inflammatory machinery in order to understand how leukocyte transcriptomics of periparturient dairy cows act to face a challenge condition. We are mindful of all limitations that our study had, but only the preliminary analysis of genes involved in recognition, immune mediation functions, migration, cell adhesion, antimicrobial strategies, inflammatory cascade, oxidative stress, and leukotriene production will allow us to plan further studies, focusing on the expression and post-translational modifications of related proteins.

## 6. Conclusions

Treatment with pegbovigrastim increased mRNA expression of most genes involved in the processes investigated including cell adhesion, migration, recognition, antimicrobial activity, and inflammation cascade. This suggested a complete activation of the immune machinery against the critical period post-partum, at least as a first response of leukocytes to transcriptional regulation. In addition, we shed light onto some differences for parity class and breed. The immune system of Simmental cows could potentially have a more acute response in early lactation, as revealed by the greater expression level of genes involved in immune system adaptation. Clearly, further efforts should be addressed to ascertain whether, when different breeds are compared for innate and adaptive immune response during the transition period, other breed-specific mechanisms are involved.

## Figures and Tables

**Table 1:** LSM of arbitrary mRNA abundance for genes related to recognition and immune mediation functions in whole blood leukocytes from Simmental and Holstein cows treated with pegbovigrastim (PEG) or saline (control (CTR) at approximately Day 7 relative to calving and within 24 h on the day of calving.

							*p*-Values					
		Breed ^1^		Parity ^2^						Group X		
Target	Group	H	S	P	M	SEM^3^	Group	Breed	Parity	Breed	Parity	Breed × Parity
CD14 *	CTR	4.33	4.11	4.20	4.25	0.17	< 0.01	0.39	0.95	0.33	0.70	0.26
	PEG	4.74	4.75	4.76	4.72							
*CD16*	CTR	89.13	111.84	104.90	96.07	21.28	< 0.01	0.71	0.58	0.06	0.96	0.33
	PEG	178.66	145.28	165.63	158.31							
MYD88 *	CTR	5.16	5.23	5.29	5.10	0.16	< 0.01	0.22	0.04	0.07	0.68	0.91
	PEG	6.38	6.03	6.34	6.06							
TLR2 *	CTR	3.95	3.74	3.93	3.76	0.13	< 0.01	< 0.01	0.02	0.40	0.59	0.64
	PEG	4.78	4.42	4.73	4.47							
TLR4 *	CTR	−1.64	−1.61	−1.38	−1.87	0.70	< 0.01	0.32	0.20	0.30	0.81	0.14
	PEG	0.31	−0.67	0.18	−0.55							

* Log10 transformed data. ^1^ H: Holstein; S: Simmental. ^2^ P: primiparous; M: multiparous. ^3^ Greatest SEM.

**Table 2:** LSM of arbitrary mRNA abundance for genes related to migration and cell adhesion functions in whole blood leukocytes evaluated 3 days after calving from Simmental and Holstein cows treated with pegbovigrastim (PEG) or with saline (CTR) at approximately Day 7 relative to calving and within 24 h the day of calving.

							*p*-Values					
		Breed ^1^		Parity ^2^						Group X		
Target	Group	H	S	P	M	SEM ^3^	Group	Breed	Parity	Breed	Parity	Breed × Parity
CCR2 *	CTR	3.58	3.60	3.53	3.65	0.18	0.27	0.53	0.36	0.66	0.91	0.49
	PEG	3.66	3.79	3.68	3.78							
*CX3CR1*	CTR	17.57	24.85	20.31	22.11	3.32	0.70	0.02	0.34	0.48	0.86	0.78
	PEG	18.31	22.36	19.02	21.65							
*ITGB2*	CTR	196.64	229.40	209.78	216.26	20.67	< 0.01	0.05	0.70	0.72	0.94	0.98
	PEG	311.46	334.14	320.59	325.01							
*ITGAL* ^4^	CTR	45.98	51.49	50.17	47.31	6.08	< 0.01	0.46	0.47	0.04	0.97	0.92
	PEG	72.02	60.33	67.79	64.57							
*TLN1*	CTR	236.39	252.30	256.96	231.73	27.71	< 0.01	0.35	0.20	0.07	0.89	0.81
	PEG	376.92	328.25	362.71	342.45							
SELL *	CTR	6.25	6.26	6.32	6.19	0.13	< 0.01	0.09	0.05	0.06	0.55	0.91
	PEG	7.29	6.97	7.25	7.02							
SELPLG * ^,4^	CTR	5.44	5.61	5.59	5.45	0.14	< 0.01	0.70	0.13	0.03	0.90	0.67
	PEG	6.48	6.23	6.43	6.27							
*CD44*	CTR	160.76	202.64	172.41	190.99	15.79	< 0.01	< 0.01	0.93	0.28	0.11	0.70
	PEG	236.07	254.13	253.50	236.70							
LGALS8 *	CTR	3.57	3.70	3.67	3.60	0.17	0.47	0.60	0.68	0.51	0.87	0.25
	PEG	3.73	3.71	3.73	3.70							

* Log10 transformed data. ^1^ H: Holstein; S: Simmental. ^2^ P: primiparous; M: multiparous. ^3^ Greatest SEM. ^4^ LSM of arbitrary mRNA abundance comparisons reported in Figure 1 and Figure 2.

**Table 3:** LSM of arbitrary mRNA abundance for genes related to antimicrobial strategies in whole blood leukocytes evaluated 3 days after calving from Simmental and Holstein cows treated with pegbovigrastim (PEG) or with saline (CTR) at approximately Day 7 relative to calving and within 24 h the day of calving.

							*p*-Values					
		Breed^1^		Parity^2^						Group X		
Target	Group	H	S	P	M	SEM^3^	Group	Breed	Parity	Breed	Parity	Breed × Parity
MMP9* ^(4)^	CTR	4.31	3.73	3.74	4.31	0.27	< 0.01	0.35	0.04	0.03	0.33	0.84
	PEG	5.26	5.49	5.27	5.48							
LTF* ^(4)^	CTR	1.57	−0.07	0.26	1.23	0.59	< 0.01	0.17	0.17	0.01	0.32	0.29
	PEG	3.78	4.28	3.95	4.10							
MPO*	CTR	0.80	1.30	0.91	1.20	0.40	< 0.01	0.13	0.47	0.74	0.73	0.29
	PEG	2.28	2.61	2.39	2.50							
LCN2*	CTR	3.41	2.87	3.05	3.24	0.41	< 0.01	0.92	0.39	0.50	0.84	0.54
	PEG	4.78	5.38	4.93	5.23							
*IDO1* ^(4)^	CTR	33.38	57.36	42.23	48.52	7.52	0.07	< 0.01	0.40	0.14	0.04	0.38
	PEG	31.65	39.98	43.32	28.31							

* Log10 transformed data. ^1^ H: Holstein; S: Simmental. ^2^ P: primiparous; M: multiparous. ^3^ Greatest SEM. ^4^ LSM of arbitrary mRNA abundance comparisons reported in Figure 3, Figure 4 and Figure 5.

**Table 4:** LSM of arbitrary mRNA abundance for genes related to the inflammatory cascade in whole blood leukocytes evaluated 3 days after calving from Simmental and Holstein cows treated with pegbovigrastim (PEG) or with saline (CTR) at approximately Day 7 relative to calving and within 24 h the day of calving.

								*p*-Values					
		Breed ^1^			Parity ^2^						Group X		
Target	Group	H	S		P	M	SEM ^3^	Group	Breed	Parity	Breed	Parity	Breed × Parity
*CASP1*	CTR	27.84	27.79		28.79	26.83	2.72	< 0.01	0.57	0.34	0.59	0.93	0.72
	PEG	35.09	33.02		34.87	33.25							
TNFRSF1A*	CTR	5.24	5.36		5.28	5.32	0.11	< 0.01	0.83	0.61	0.16	0.35	0.84
	PEG	6.08	5.99		6.09	5.98							
IL1B* ^(4)^	CTR	4.22	4.72		4.66	4.28	0.24	< 0.01	0.48	0.02	0.02	0.92	0.55
	PEG	5.89	5.63		5.97	5.55							
IL1R*	CTR	3.31	3.44		3.38	3.37	0.12	< 0.01	0.24	0.83	0.71	0.95	0.75
	PEG	3.96	4.02		4.00	3.98							
IL8*	CTR	3.23	2.80		3.00	3.03	0.29	0.02	0.13	0.67	0.55	0.55	0.61
	PEG	2.62	2.43		2.63	2.42							
IL18*	CTR	2.48	2.43		2.58	2.33	0.45	< 0.01	0.25	0.04	0.31	0.22	0.24
	PEG	4.93	4.26		5.11	4.08							
*IRAK1*	CTR	41.60	40.30		42.11	39.79	3.32	< 0.01	0.65	0.24	0.90	0.87	0.51
	PEG	58.82	58.08		59.99	56.91							
*TNF*	CTR	9.30	10.97		9.88	10.39	0.82	0.02	< 0.01	0.93	0.55	0.41	0.39
	PEG	7.66	10.00		9.04	8.62							
NLRP3*	CTR	3.46	3.44		3.50	3.40	0.13	< 0.01	0.20	0.30	0.28	0.98	0.51
	PEG	4.29	4.07		4.23	4.13							
*S100A8*	CTR	1235.74	819.56		951.22	1104.09	386.69	< 0.01	0.29	0.99	0.62	0.57	0.83
	PEG	2467.31	2314.12		2464.71	2316.71							
*RPL13A*	CTR	931.33	1089.30		1063.98	956.66	96.06	< 0.01	0.05	0.13	0.22	0.91	0.93
	PEG	752.68	878.89		869.84	761.73							

* Log10 transformed data. ^1^ H: Holstein; S: Simmental. ^2^ P: primiparous; M: multiparous. ^3^ Greatest SEM. ^4^ LSM of arbitrary mRNA abundance comparisons reported in Figure 6.

**Table 5:** LSM ± SEM of arbitrary mRNA abundance for genes related to oxidative stress in whole blood leukocytes evaluated 3 days after calving from Simmental and Holstein cows treated with pegbovigrastim (PEG) or with saline (CTR) at approximately Day 7 relative to calving and within 24 h the day of calving.

								*p*-Values					
		Breed ^1^			Parity ^2^						Group X		
Target	Group	H	S		P	M	SEM ^3^	Group	Breed	Parity	Breed	Parity	Breed × Parity
SOD1 *	CTR	4.08	4.11		4.16	4.03	0.11	0.29	0.55	0.10	0.35	0.94	0.34
	PEG	4.07	3.96		4.08	3.95							
SOD2 *	CTR	5.48	5.60		5.66	5.42	0.28	< 0.01	0.49	0.07	0.17	0.64	0.95
	PEG	7.25	6.89		7.27	6.87							
*ALOX5*	CTR	17.61	23.71		17.40	23.92	4.28	< 0.01	< 0.01	0.06	0.23	0.76	0.60
	PEG	22.30	35.48		26.52	31.26							
*ALOX15*	CTR	23.83	34.06		27.75	30.14	5.40	0.02	0.09	0.68	0.30	0.82	0.77
	PEG	18.92	21.37		19.79	20.50							

* Log10 transformed data. ^1^ H: Holstein; S: Simmental. ^2^ P: primiparous; M: multiparous. ^3^ Greatest SEM.

## Supplementary Materials

The following are available online at https://www.mdpi.com/2076-2615/10/4/621/s1: Figure S1: Changes of the whole blood leukocytes gene expression in the KEGG TOLL-LIKE RECEPTOR signaling pathway in PEG compared with CTR cows, regardless the breed; Figure S2: Changes of the whole blood leukocytes gene expression in the KEGG NOD-LIKE RECEPTOR signaling pathway in PEG compared with CTR cows, regardless the breed; Figure S3: Changes of the whole blood leukocytes gene expression in the KEGG NF-KAPPA B signaling pathway in PEG compared with CTR cows, regardless the breed; Figure S4: Changes of the whole blood leukocytes gene expression in the KEGG CELL ADHESION molecules in PEG compared with CTR cows, regardless the breed.
