# Supplementary material for: Pegbovigrastim Treatment around Parturition Enhances Postpartum Immune Response Gene Network Expression of whole Blood Leukocytes in Holstein and Simmental Cows
Source: Animals (Basel). 2020 Apr 3;10(4):621. doi: 10.3390/ani10040621 (PMC7222845; doi:10.3390/ani10040621)
Supplement: Supplementary file 1 [file animals-10-00621-s001.pdf]

Supplementary File

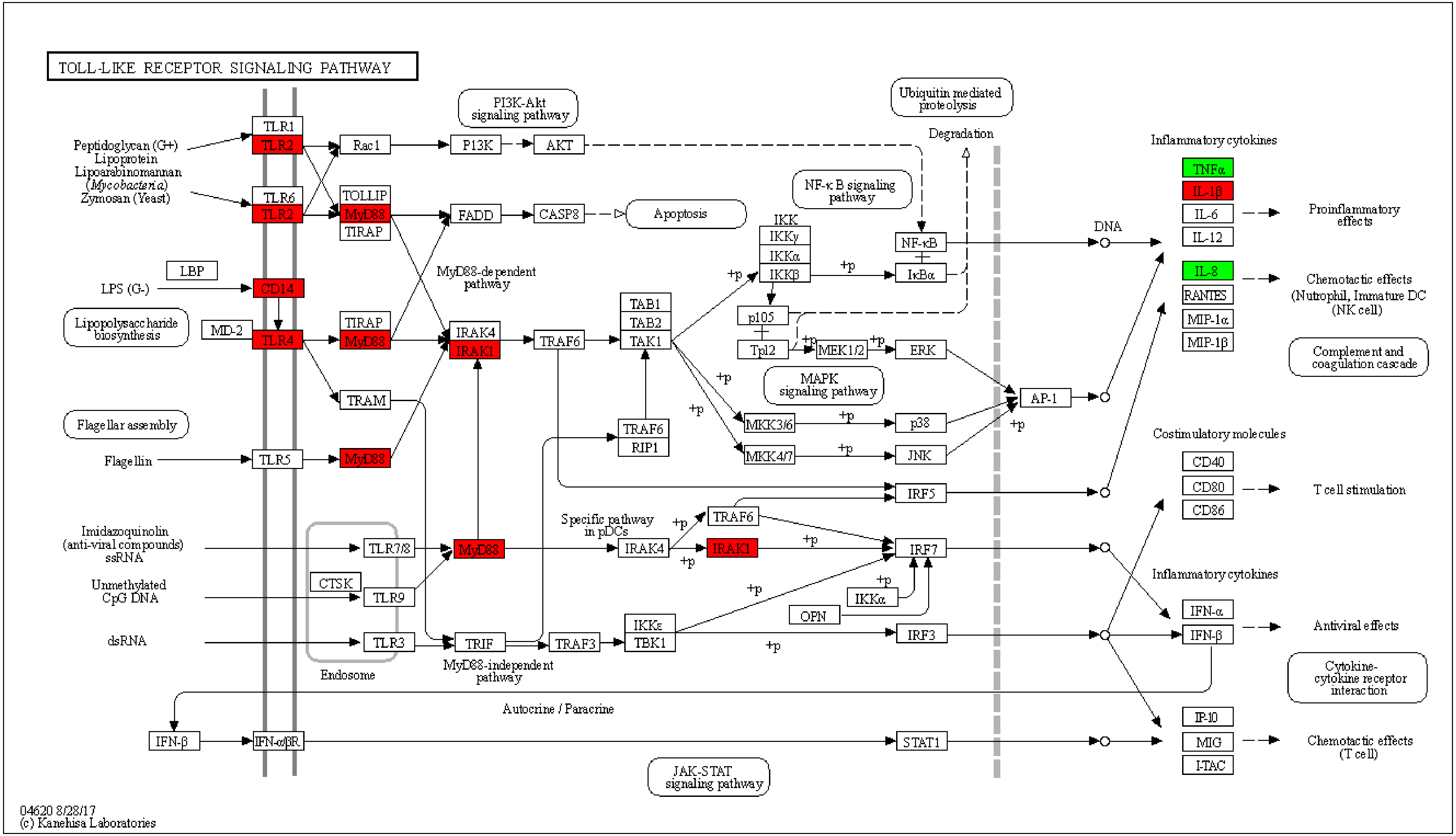

Figure S1. Changes of the whole blood leukocytes gene expression in the KEGG TOLL-LIKE RECEPTOR signaling pathway in PEG compared with CTR cows, regardless the breed. Red boxes are genes with increased expression in PEG cows, whereas green boxes are genes with reduced expression in PEG cows.

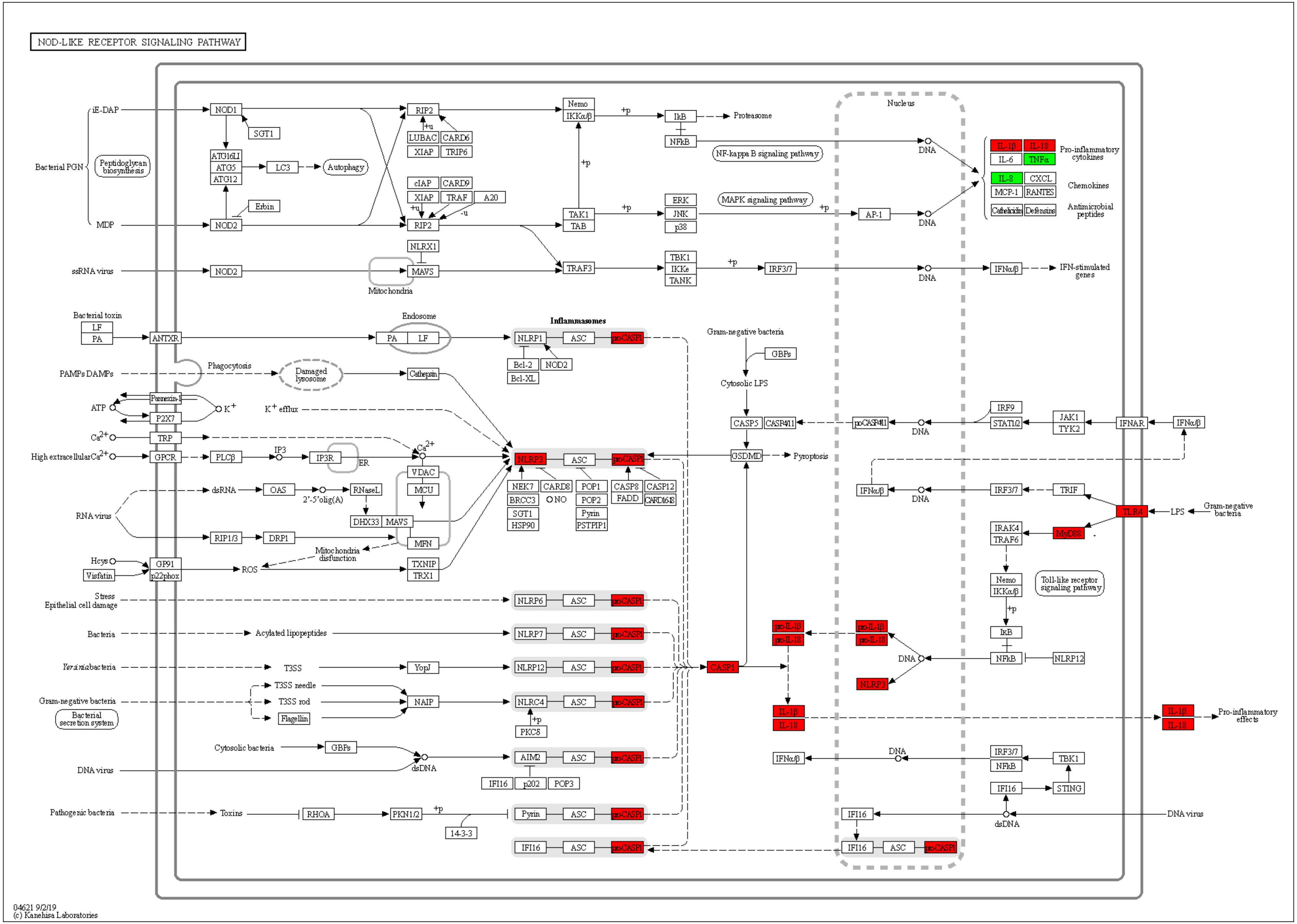

Figure S2. Changes of the whole blood leukocytes gene expression in the KEGG NOD-LIKE RECEPTOR signaling pathway in PEG compared with CTR cows, regardless the breed. Red boxes are genes with increased expression in PEG cows, whereas green boxes are genes with reduced expression in PEG cows

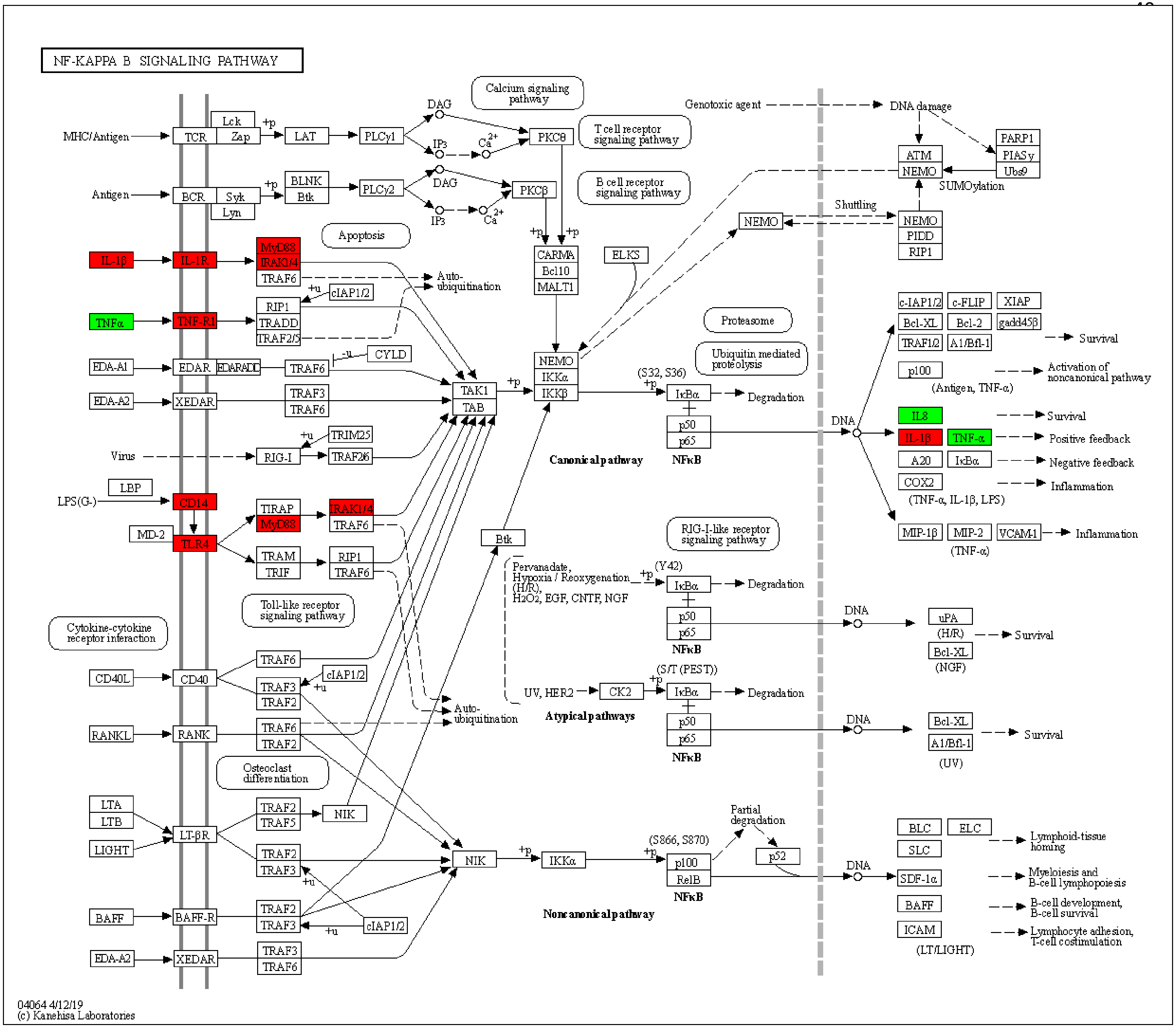

Figure S3. Changes of the whole blood leukocytes gene expression in the KEGG NF-KAPPA B signaling pathway in PEG compared with CTR cows, regardless the breed. Red boxes are genes with increased expression in PEG cows, whereas green boxes are genes with reduced expression in PEG cows.

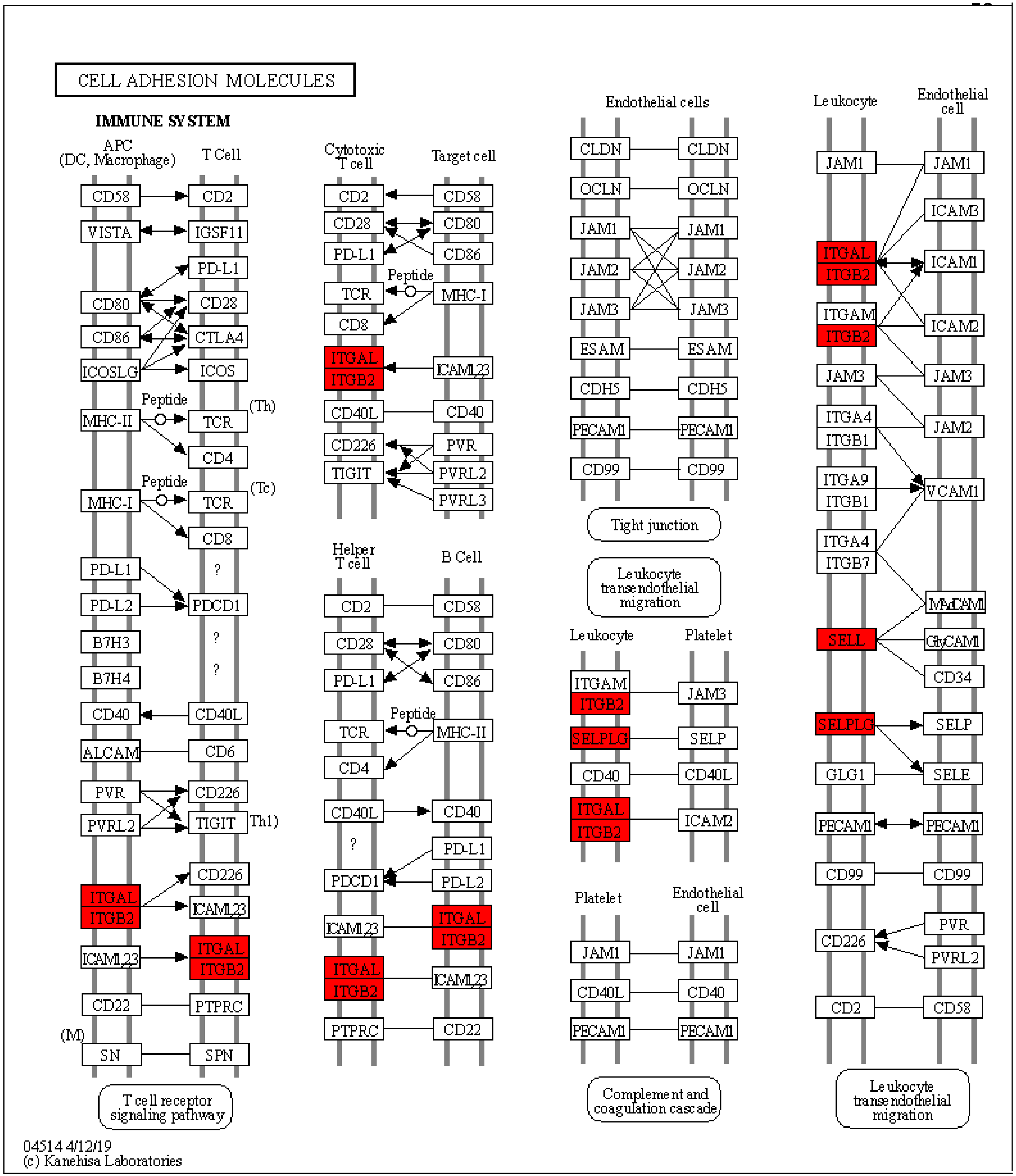

Figure S4. Changes of the whole blood leukocytes gene expression in the KEGG CELL ADHESION molecules in PEG compared with CTR cows, regardless the breed. Red boxes are genes with increased expression in PEG cows, whereas green boxes are genes with reduced expression in PEG cows.
